# Ketogenic diet effects on 52 children with pharmacoresistant epileptic encephalopathy: A clinical prospective study

**DOI:** 10.1002/brb3.973

**Published:** 2018-04-18

**Authors:** Qiong Wu, Hua Wang, Yu Ying Fan, Jun Mei Zhang, Xue Yan Liu, Xiu Ying Fang, Feng Hua Yang, Qing Jun Cao, Ying Qi

**Affiliations:** ^1^ Department of Pediatric Neurology Shengjing Hospital of China Medical University Shenyang China; ^2^ Department of Functional Neurology Office Shengjing Hospital of China Medical University Shenyang China; ^3^ Department of Radiology Shengjing Hospital of China Medical University Shenyang China

**Keywords:** children, ketogenic diet, pharmacoresistant epileptic encephalopathy

## Abstract

**Objective:**

To evaluate the clinical impact of ketogenic diet (KD) on children with pharmacoresistant epileptic encephalopathy.

**Methods:**

In all, 52 children with pharmacoresistant epileptic encephalopathy that diagnosed in our hospital from July 2012 to June 2015 were selected, including West syndrome 38 cases, Lennox–Gastaut Syndrome 7 cases, Doose Syndrome 1 case, and Dravet syndrome 6 cases, and the effect, compliance, adverse reactions, electroencephalogram (EEG), and cognitive function were analyzed. Modified Johns Hopkins protocol was used to initiate KD, and Engel scale was used to evaluate the effect, and evaluated the effect of KD on the cognition, language, and motor function.

**Results:**

At 12 weeks of KD treatment, the patients achieved I, II, III, and IV grade effect were accounted for 26.9% (14/52 cases), 17.3% (9/52 cases), 11.5% (6/52 cases), and 44.2% (23/52 cases), respectively, according to Engel scale. KD has different effect on different epileptic syndromes, best effect on Doose syndromes of 100%, and better effect on West syndrome with the effect rate of 57.9%, and the total effect number was 22 cases. The reduction of epileptiform discharges in the awake state before KD treatment was correlated with the seizure time after 3 months of KD treatment (*r *=* *.330, *p* = .017). The cognitive function of 23 patients was improved, 12 patients had language improvement, and the motor function was improved in 10 patients. In all, 23 patients had adverse reactions, and all patients were tolerated and improved.

**Conclusion:**

KD has certain effect on children with pharmacoresistant epileptic encephalopathy, and it can reduce interictal epileptic discharge frequency, and improve the background rhythm of EEG. The reduction of epileptiform discharges in awake state is in favor of the reduction of seizures frequency, thus increasing the efficacy, and improve the cognitive function, language, and motor function to varying degrees, combined with less adverse reaction, which is worthy of clinical application.

## 1 INTRODUCTION

Pharmacoresistant epileptic encephalopathy in children refers to the regular treatment with two or more than two antiepileptic drugs still cannot completely control the onset of epilepsy (Farrell, Wirrell, & Whiting, 2006; Kwan et al., 2010), also known as drug‐refractory epilepsy, about 20%–30% of epilepsy children will eventually develop for refractory epilepsy (Zupec‐Kania & Spellman, 2008). Children epileptic encephalopathy is the cerebral disease with the main clinical characteristics of frequent seizures of epilepsy, progressive neurological decay, interictal electroencephalogram (EEG) persistent epileptic discharge, difficult in the treatment with traditional antiepileptic drugs, and poor prognosis, and it is called pharmacoresistant epileptic encephalopathy in children. At present, the treatment of pharmacoresistant epileptic encephalopathy in children is new antiepileptic drug therapy, surgery, vagus nerve stimulation, deep brain stimulation, transcranial magnetic stimulation, ketogenic diet (KD), etc. KD therapy for the treatment of pharmacoresistant epileptic encephalopathy in children at home and abroad has achieved a certain curative effect. KD is a high proportion of fat, low carbohydrate, adequate protein diet program, and the body supply energy mainly rely on fat instead of carbohydrates. By simulating hunger state, the ketone body in its fat metabolism progress can be used as another kind if energy supply source, can generate antiepileptic effect. KD was first reported by Wilder in 1921, it confirmed that KD has an exact curative effect in the treatment of refractory epilepsy. At present, more than 70 countries around the world have carried out KD therapy, and most of epilepsy center in the United States make KD as one of the conventional treatment methods for refractory epilepsy (Kossoff & McGrogan, 2005). In China, KD has gradually carried out for the treatment of refractory epilepsy. In this study, we summarized the effect of KD in the treatment of children with refractory epilepsy, as well as the compliance, adverse reactions, EEG, and its impact on cognition.

## 2 MATERIALS AND METHODS

### 2.1 General materials

According to the criteria of the diagnosis of pharmacoresistant epileptic encephalopathy of International Union Against Epilepsy, 52 children selected from the Department of Outpatients of Shengjing Hospital of China Medical University from July 2012 to June 2015 were all consistent with the features of pharmacoresistant epileptic encephalopathy (Zupanc, 2009). In all, 30 males and 22 females, aged 3 months to 7 years, course of disease >2 months were selected. A total of 65 children were selected in the initial group, of which 8 cases of children refused to KD, 3 cases cannot insist due to diarrhea after KD treatment, 1 case gave up because of the significant nausea and vomiting after KD treatment, and 1 case stopped treatment due to the initial treatment of pneumonia, and a total of 13 cases gave up in the early treatment. The remaining 52 cases were treated for more than 12 weeks and included in this study. The parents of the included children were signed the informed consent. Classification according to syndrome: 38 cases of infantile spasms, of which 1 case had family history of epilepsy, 5 cases had the history of neonatal hypoxic ischemic brain disease, and 2 cases had tuberous sclerosis; 7 cases of Lennox–Gastaut syndrome, of which 2 cases had family history of epilepsy, 1 cases had family history of febrile seizures, 1 case had history of craniocerebral trauma, and 2 cases had viral encephalitis sequelae; 1 case of Doose syndrome; 6 cases of Dravet syndrome, of which 3 cases had family history of febrile seizures. All the 52 patients received psychomotor developmental assessment, and result showed varying degrees of psychomotor development behind the same age children. Imaging examination: the patients received imaging examination, and 16 cases were abnormal, including 2 cases of brain CT showed periventricular multiple calcification (it considered as tuberous sclerosis through skin discoloration), 3 cases of MRI showed ventricular dilatation, 1 case of MRI showed Dandy–Walker variation, 10 cases of cerebral hypoplasia, and the remaining 36 cases had no apparent abnormalities (5 cases received brain CT and 31 cases received brain MRI).

### 2.2 Inclusion criteria

All the patients were diagnosed as pharmacoresistant epileptic encephalopathy; all patients had taken two or more than two kinds of antiepileptic drugs, and there still frequent seizures after regular treatment (1 month seizure > 4 times); no patients had received KD treatment previously; excluded fat metabolic diseases, immune deficiency diseases and digestive system, cardiovascular system, respiratory system, digestive system, and other serious diseases. All of the families of the 52 patients agreed to KD treatment, and signed the informed consent, and received regular follow‐up. All patients received KD treatment for more than 12 weeks. This study has been approved by the ethics committee of the hospital.

### 2.3 Preparation of KD

Nutritionists prepared KD in accordance with the modified Johns Hopkins program, and only KD treatment was conducted (Kossoff & Drward, 2008). The basic principles are as follows: (1) the calorie is about 75% of the total calories recommended by the age and body mass of children, for the children with large activity, the calorie will be increased moderately; (2) all children without strict fasting, and received KD directly (Health ketone products provided by Guangzhou Ketone Company); (3) the amount of liquid intake is not limited with a small number of drinking water, but no more than 120 ml per time; (4) added protein, minerals, vitamins, and dietary fiber.

### 2.4 Treatment methods

Gas chromatography–mass spectrometry and high‐performance liquid chromatography–tandem mass spectrometry were conducted to exclude the metabolic diseases, and then the patients received blood routine, urine routine, dung conventional, blood lipid, liver function, electrocardiogram, electroencephalogram, and urinary system ultrasonography. All the patients received KD treatment directly after using antiepileptic drugs without fasting. The fat and non‐fat ratio was increased from 0.5:1.0 to 4.0:1.0 within 1–2 months according to the specific circumstances of patients, then prepared KD recipe based on the “KD catering software” that fit the Chinese eating habits, the KD ratio was selected according to the situation of the patients (0.5:1.0, 1.0:1.0, 1.5:1.0, 2.0:1.0, 2.5:1.0, 3.0:1.0, 3.5: 1.0, 4.0:1.0) to ensure the daily protein and calorie requirements. Each EI contained calorie 251.2–334.9 KJ/kg and protein 1–2 g/kg, and intake the daily calorie through 4–6 times. Meanwhile, the vital signs, blood glucose, ketone changes, and statistics the seizures of epilepsy and the related adverse reactions were monitored.

### 2.5 Fully digital video electroencephalogram (VEEG) monitoring

According to different age, VEEG is monitored at 2–5 hr before sleep. EEG9100 VEEG instrument produced by Japan Optoelectronics Company was used. In accordance with the international 10–20 system installed 19‐guide to record the electrode, the bilateral ear electrode was used as the reference electrode, and additional electrocardiogram (ECG), electromyogram (EMG), and sphenoid electrode were used. The electrode was the silver plate electrode, coated with solid electrode paste attached to the head, elastic cap was used to further fix outside. Dual camera was used to monitor the seizure condition (one of the camera target on the head of the patients, and the other one target on the whole body of the patients), then recorded with the reference guide mode. During record, the patients should complete open and close eyes, hyperventilation, and flash‐induced special test within 0.5 hr, and at least keep awake for 1 hr and state sleep for 2 hr. The monitoring process should contain at least one complete sleep cycle. When monitoring, the relatives who familiar with the patients should be on the scene to distinguish whether the seizures is similar with the previous seizures. For the pseudo‐seizure patients, corresponding implied induction test should be conducted. Marked various states and events during and after monitoring, and when playback, analyzed with the video synchronously step by step, and could change the recorded digital EEG lead connection mode, amplitude voltage, and filtering if necessary. EEG diagnosis was based on the diagnostic criteria of “Clinical EEG.”

### 2.6 Observation of the curative effect

(1) Determined the onset time of KD for the treatment of children with pharmacoresistant epileptic encephalopathy: record the seizures of epilepsy and the average number of seizures 1 month before KD treatment was considered the basic seizure frequency. During KD treatment, recorded the seizure frequency and form changes, the guardian record the seizure journal, and timely follow‐up to determine the first time of epilepsy remission. (2) Evaluation of KD efficacy: referred to Engel classification (Engel, 1996). Grade I: complete remission after treatment; Grade II: only a rare epilepsy that affect the function seizure (remission 90%–100%); Grade III: the seizure of epilepsy has been improved (remission 50% to <90%); Grade IV: no significant improvement (remission <50%). The seizure time decreased more than 90% after treatment, included completely controlled, was considered as markedly (including Grade I and Grade II), 90%–50% was considered as effective (Grade III), and <50% was invalid (Grade IV). In this study, the seizure of epilepsy decreased ≥50% was considered effective. The seizure frequency and form at 4, 12, and 24 weeks after KD treatment is recorded to determine the efficacy level. (3) Evaluation the effect of KD treatment on EEG: It was recommended that the patients should be reviewed the 4‐hr EEG at 4 and 12 weeks after KD treatment, and the patients should receive ≥4 hr video EEG monitoring before treatment and at 3 months after treatment, respectively. First, the occipital background rhythm and interictal Spike‐Index (SI) under awake and quiet state were assessed. SI is the average number of spike discharges within 1 s, the calculation method: randomly selected 100S EEG in awake and quiet state (no artifact), and calculated the number of spikes (*n*), SI = *n*/100. The changes of background rhythm interictal epileptiform discharge frequency are evaluated. Finally, the epileptiform discharge index at 1 hr after awaking and sleeping [total time of epileptiform release (*s*)/total observation time (*s*) × 100%] is calculated, respectively, and the corresponding epileptiform discharge index during awake period and sleep period. (4) Evaluation on the impact of KD treatment on cognitive function: Gesell Development Scale was used to evaluate the cognitive function, language, motor ability of patients after treated with KD for over 12 weeks. (5) Evaluation of adverse reactions: statistics the adverse reaction during treatment through the description on the general information of the patients by the parents and the testing.

### 2.7 Statistical analysis

SPSS19 was used to analyze the data. Spearman rank correlation analysis was used to analyze the relationship between the decreasing of epileptiform discharge index before KD treatment and 1 month after KD treatment and the decreasing of seizures at 3 months after KD treatment. *p* < .05 was considered statistically significant.

## 3 RESULTS

### 3.1 Clinical seizures, drug treatment, treatment efficacy, and onset time within 1 month after KD treatment

Seizure frequency was ranged from 1 time every 2–3 days to more than 20 times per day, infantile spasms seizure 1 time per day. Six cases had the average number of seizures ≤5 times per day, 13 cases had the average number of seizures 6–10 times per day, 21 cases had the average number of seizures 10–20 times per day, and 12 cases had the average number of seizures ≥20 times per day. All the 52 patients were treated with more than two kinds of antiepileptic drugs for conventional treatment, but the effect was not obvious, and among the patients, 15 patients were treated with two kinds of antiepileptic drugs, 17 patients received three kinds of antiepileptic drugs, and 20 patients were treated with more than four kinds of antiepileptic drugs. The number of the patients that adhere to KD treatment for 4, 12, 24, and 48 weeks were 52 cases (100%), 52 cases (100%), 41 cases (78.8%), and 29 cases (55.8%), respectively, which had the high retention rate. After KD treatment, the efficiency showed an increasing trend with treatment time prolonging. It was effect in 29 cases after treated for 12 weeks, and all start effect within 1–2 weeks after treatment, 15 cases start effect within 1 week, and 13 cases start effect within 2 weeks; after treated for 12 weeks, 14 cases had no seizures, markedly in 9 cases, effective in 6 cases, and the total effective rate was 55.8%, invalid in 23 cases (Table 1).

**Table 1:** Efficacy of KD in the treatment of pharmacoresistant epileptic encephalopathy [*n* (%)]

Syndrome	*N*	Treatment time (weeks)	Engel classification				Effective (%)
			I grade	II grade	III grade	IV grade	
Infantile spasm	38	4	11	5	4	18	20 (52.6)
	38	12	11	7	4	16	22 (57.9)
	34	24	11	7	4	12	22 (64.7)
Lennox–Gastaut syndrome	7	4	1	0	1	3	2 (28.6)
	7	12	1	1	1	4	3 (42.9)
	2	24	1	0	0	1	1 (50.0)
Doose syndrome	1	4	1	0	0	0	1 (100)
	1	12	1	0	0	0	1 (100)
	1	24	1	0	0	0	1 (100)
Dravet syndrome	6	4	1	0	1	4	2 (33.3)
	6	12	1	1	1	3	3 (50.0)
	4	24	1	1	0	2	2 (50.0)
Total case	52	12	14	9	6	23	29 (55.8)

### 3.2 Efficacy of KD on epilepsy syndrome

The information of the patients after adhering to KD treatment for 4, 12, and 24 weeks are shown in Table 1. After treated for 12 weeks, KD had different effect in the treatment of different epilepsy syndrome, of which Doose syndrome had the best effect, 1 case and no seizures, but due to the less included case, the efficacy remains to be assessed; followed by infantile spasms, 11 cases (totally 38 cases) had no seizures, and the total effective rate was 57.9%; Dravet syndrome in 6 cases, 3 cases effective and 3 cases invalid; Lennox–Gastaut in 7 cases, and 3 cases had no seizures, the total effective rate was 42.9% (Table 1).

### 3.3 KD improved EEG

**Figure 1:**
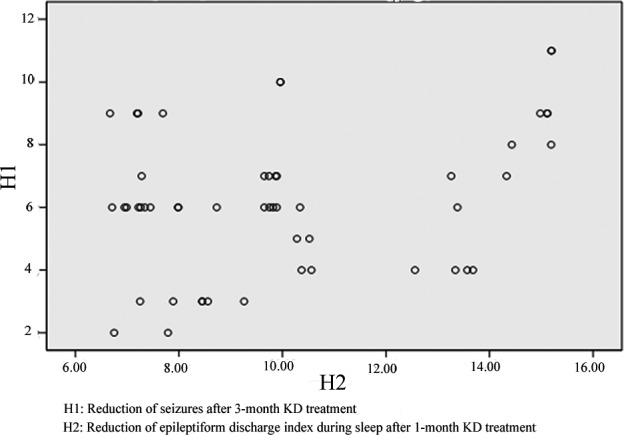
Correlation between reduction in epileptiform discharge index during sleep after 1‐month KD treatment and the reduction in the seizure after 3‐month KD treatment

EEG showed a significant improvement in background rhythms in 19 patients at 12 weeks of treatment, and spike (sharp) wave index was significantly reduced in 26 patients (*p* < .05, reduction > 30%, Table 2). There was correlation between the reduction in epileptiform discharge index during sleep after 1‐month KD treatment and the reduction in the seizure after 3‐month KD treatment (*r* = .330, *p* = .017 < .05, Figure 1). There was no correlation between the reduction in epileptiform discharge index during awake after 1‐month KD treatment and the reduction in the seizure after 3‐month KD treatment (*r* = .243, *p* = .082 > .05).

**Table 2:** Background rhythm and spike wave discharge index changes after KD treatment [*n* (%)]

Treatment duration (months)	Background rhythm frequency			Spike wave discharge index			
	Increased ≥ 2 time/s	Increased 1–2 time/s	Increased < 1 time/s	Decreased ≥ 75%	Decreased ≥ 50%	Decreased ≥ 30%	Decreased < 30%
1	5	9	38	11	5	4	31
3	7	12	33	12	8	6	26

### 3.4 KD promote cognitive improvement, language progress, and motor development

The cognitive function was evaluated by Gesell development scale. The motor function, language ability, and cognitive function of some children after KD were improved, but the improvement was too small to be statistically significant. Among them, 23 cases had the improvement in cognitive function, of which 14 had no seizures after treated for 12 weeks, 6 cases had rare seizures, seizures in 3 cases were improved (the seizures reduced 50%–90%); 12 cases had the improved in language, of which 7 cases had no seizures after treated for 12 weeks, 4 cases had rare seizures (the seizures reduced 90%–100%), and 1 case achieved significant improvement after seizure (the seizure reduced 50%–90%); 10 cases had the improvement in motor function, of which 5 cases had no seizures after treated 12 weeks, rare seizures in 4 cases (the seizures reduced 90%–100%), and 1 case achieved significant improvement after seizure (the seizure reduced 50%–90%).

### 3.5 Adverse reaction of KD

In this study, the adverse reactions of KD on children are not serious. During KD treatment, 23 cases had 1–3 kinds of adverse reactions, of which 20 cases had varying degrees of digestive symptoms, and during this period, 4 cases sustained this symptom, 11 cases had asymptomatic hypoglycemia symptoms, and 12 cases had the symptom of significant sleep increasing within 1 week of onset, and 1 case had abnormal liver function. After symptomatic treatment, the adverse reactions in most patients could subside, and did not affect KD treatment.

## 4 DISCUSSION

Epileptic encephalopathy has a poor response to antiepileptic drugs, and is thus considered to be pharmacoresistant epileptic encephalopathy, which accounts for 20%–30% of epilepsy cases. Although antiepileptic drugs continue to be developed, the incidence of epileptic encephalopathy has not changed. With the exception of antiepileptic drugs for the treatment of pharmacoresistant epileptic encephalopathy in children, the treatment methods include KD, surgery, and vagus nerve stimulation therapy. As a result of the low cost of treatment, low risk, and definite curative effect, KD therapy was widely used domestically and internationally before antiepileptic drugs were available. At present, KD therapy and related treatment methods have been shown to be effective in the treatment of pharmacoresistant epileptic encephalopathy in children (Winesett, Bessone, & Kossoff, 2015). The international guidelines for the treatment of pharmacoresistant epileptic encephalopathy in children by KD were published in 2009, and the updated paper published by the International Union Against Epilepsy in 2015 indicated that KD is a safe and effective method for the treatment of pharmacoresistant epileptic encephalopathy in children (van der Louw et al., 2016).

The antiepileptic mechanism underlying KD is unclear, yet the use of ketones as the major source of energy in the brain and calorie restriction appears to be important for the effects of KD (Masino & Rho, 2012). From a historical point of view, the mechanism of action of KD is considered to be the antiepileptic effect of ketones because the production of ketones is a significant feature of the diet. The ketones produced by KD include three species (acetoacetate, acetone, and beta‐hydroxybutyrate [BHB]). Acetoacetate and acetone have an antiepileptic effect in a variety of acute exacerbation rat models, but BHB does not show antiepileptic effects. It is surprising that the level of BHB is often clinically associated with the control of epilepsy in children. Recent studies using different levels of ketones, and experiments have shown that there is insufficient evidence to prove that the efficacy of KD is derived from the antiepileptic effect of ketones. In addition, the level of ketones does not correspond with the antiepileptic effect (Masino & Rho, 2012). Another study showed that ketones do not have an effect on the main ion channels (GABA A, ionotropic glutamate receptor, or the voltage‐gated sodium channels [the antiepileptic drugs are commonly used as a target for this ion channel]) (Thio, Wong, & Yamada, 2000). Existing evidence suggests that BHB can reduce the degradation of GABA, and increase the concentration of inhibitory neurotransmitters (Suzuki et al., 2009). Interestingly, low glycemic index treatment, as a variant of the KD, does not require high levels of blood ketones to achieve the desired effect. In addition, glucose pathway inhibitors also have antiepileptic effects, suggesting that calorie restriction and reduced glucose availability may be an important mechanism underlying the antiepileptic effect of KD (Stafstrom et al., 2009). Although the mechanism of action underlying KD is complex, the efficacy of KD is broader than most of the anticonvulsant drugs. In this context, “metabolic‐based treatment” could be a new class of anticonvulsant therapy (Kossoff, Zupec‐Kania, & Rho, 2009), and a study on the mechanism underlying KD has potential benefits for children. Bough, Valiyil, Han, and Eagles (1999)) found that the efficacy of KD is age‐dependent; thus, the younger the children, the more significant the antiepileptic effect. Therefore, KD is mainly used in pediatrics.

KD had a good effect on a variety of seizures (Beniczky, Jose Miranda, Alving, Heber Povlsen, & Wolf, 2010; Hallböök, Köhler, Rosén, & Lundgren, 2007; Henderson, 2006; Keene, 2006; Remahl, Dahlin, & Amark, 2008), and KD has been shown to reduce seizure occurrence by 50%, with some children achieving complete control (Winesett et al., 2015). Studies have shown that KD has varying degrees of effectiveness for infantile spasms (Hong, Turner, Hamdy, & Kossoff, 2010), Dravet syndrome (Laux & Blackford, 2013), and Doose syndrome (Bergqvist, 2012), and is relatively effective on infantile spasms. This result provided a good theoretical basis for this study. Kossoff, Zupec‐Kania, & Amark, et al. (2009) suggested that KD has special effects on some epilepsy syndromes, such as myoclonic‐atonic epilepsy, Dravet syndrome, and West syndrome (especially combined with tuberous sclerosis). Others have observed that a KD has a significant effect on Dravet and Doose syndromes. Caraballo et al. (2006) reported the results of KD therapy in 11 patients with refractory myoclonic‐astatic epilepsy. Two patients (2/11) were seizure‐free, 2 (2/11) had a 75%–99% decrease in seizures, and the remaining 2 children (2/11) had a 50%–74% decrease in seizures (Caraballo et al., 2006). Greater than one‐half of children had a 50% reduction in seizures, with a significant proportion of those having no seizures (Caraballo et al., 2006; Kilaru & Bergqvist, 2007; Korff et al., 2007). It can be concluded that KD has gradually been recognized as beneficial in the treatment of Dravet syndrome, West syndrome, and Doose syndrome. The results of this study showed that the efficiency of KD increased with time. In all, 52 children with epileptic encephalopathy were treated with KD for at least 12 weeks; 41 children were treated for 24 weeks, and 29 children were treated for 48 weeks. Complete control of seizures occurred in 12 patients, and the number of seizures in 29 patients was reduced by >50%; KD was shown to be effective within 2 weeks of beginning treatment. At 12 weeks of KD treatment, among 52 children, Engel I grade accounted for 26.9% (14/52), Engel II grade accounted for 17.3% (9/52), Engel III grade accounted for 11.5% (6/52), and Engel IV grade accounted for 44.2% (23/52). When treated for 12 weeks, different epilepsy syndromes respond differently to KD. Doose syndrome has the best response to KD, but due to the small number of cases, the efficacy needs further evaluation. Infantile spasms respond better to KD; 11 of 38 children had no seizures, and the seizure activity of 57.9% (22/38) children was decreased by >50%. KD also had a good therapeutic effect in the treatment of Dravet syndrome. During the treatment of Lennox–Gastaut syndrome in 7 children, 3 had no seizures and the effective rate was 42.9%. The onset of the effect of KD was rapid in the treatment of epileptic encephalopathy in children, and this was effective in nearly one‐half of the children. This conclusion was consistent with international research, especially in the treatment of Doose and West syndromes. The effect was relatively good with a satisfactory clinical response, which is worthy of application for clinicians.

KD cannot only effectively alleviate clinical seizures in children with epilepsy, but can also significantly improve the EEGs of children with epilepsy. Hallböök et al. (2007) found that the interictal epileptic discharge frequency 3 months after KD treatment was significantly decreased compared with pre‐treatment. Remahl et al. (2008) compared the 24‐hr EEG of 23 children who were treated with KD before and after treatment. In addition to the 65.2% (15/23) of patients with a decrease in interictal epileptic discharge frequency, the background rhythm was more normal, and these changes were shown on EEGs (Remahl et al., 2008). Even the patients who had poor efficacy after KD treatment, the changes in EEGs were apparent (Remahl et al., 2008). Dressler et al. (2010) also reported a significant improvement in interictal epileptic discharge frequency and the background rhythm in the EEGs after 6 months of KD treatment. Kessler, Gallagher, Shellhaas, Clancy, and Bergqvist (2011) observed 48 children treated with KD, and suggested that among the patients with a significant decrease in the discharge index at 1 month after KD treatment, the control of convulsions after 3 months was significantly improved. Ebus et al. (2014) studied the EEGs of 34 children with pharmacoresistant epileptic encephalopathy, and found that there was a correlation between the reduction in the epileptiform discharge index during sleep after 6 weeks of KD treatment and the decrease in clinical seizures after 6 months of KD treatment. The efficacy in patients with a decrease >30% in the epileptiform discharge index during sleep after 6 weeks of KD treatment was better (Ebus et al., 2014). In this study, the data showed that prolonging the KD treatment time significantly improved the patients' background rhythm. After treatment for 12 weeks, the EEGs of 19 children had improved background rhythms, and the interictal spike (cusp) wave index was decreased (>30%) in 26 children. There was a correlation between the reduction in the epileptiform discharge index during sleep after 1 month of KD treatment and the decrease in seizure after 3 months of KD treatment (*r* = .330, *p* = .017). We suggest that the early decrease in the epileptiform discharge index during sleep is conducive to the reduction in seizures of epileptic encephalopathy, thereby enhancing the efficacy. There are several studies which have confirmed that the reduction in early onset is more common than a reduction in relayed onset (Bergqvist, Schall, Gallagher, Cnaan, & Stallings, 2005; Coppola et al., 2002; Mosek, Natour, Neufeld, Shiff, & Vaisman, 2009). Some believe that such changes in EEGs occur because KD can produce antiepileptic‐like favorable electrophysiologic effects on the brain (Cantello et al., 2007; Kessler et al., 2011), and this effect can also be demonstrated in the brain electrophysiologic characteristics of patients with a poor response to KD treatment, indicating that KD has a positive effect on the central nervous system and cortical neurons, thus suggesting that KD can be used to treat epileptic electrical status, such as epilepsy with continuous spike‐waves during slow sleep and acquired epileptic aphasia to help improve cardiac function in children.

The basic purpose of KD treatment is to control seizures, reduce, and discontinue using medications, and to improve the cognitive ability of children. It has been shown that during KD treatment of children with epilepsy, a decrease in seizure frequency significantly improves the lives of children, and children actively communicate with their parents (Peuscher et al., 2012). The follow‐up results showed that after KD treatment, some children had improvement in motor function, language ability, and cognitive function. In all, 23 patients had cognitive improvement, including 14 without seizures, 6 with infrequent seizures, and 3 with significant improvement 12 weeks after treatment. In all, 12 patients exhibited improvement in language ability, including 7 without clinical attacks, 4 with sporadic seizures, and 1 with a significant improvement 12 weeks after treatment. In all, 10 patients had motor function improvement, including 5 without seizures, 4 with rare seizures, and 1 had significant improvement 12 weeks after KD treatment, but the degree of improvement in cognitive function was too small to reach statistical significance. This result is similar to the international study conducted by Lambrechts et al. (2013). KD treatment is a relatively safe treatment method, which can play a role in cognitive and motor function of children, and a large number of tests have confirmed that KD can control the onset of pharmacoresistant epileptic encephalopathy and improve cognition, which may be reflect a reduction in memory impairment caused by seizures in KD‐treated children (Jiang et al., 2016). The mechanism underlying KD treatment may be related to neuroprotection (Hallböök, Ji, Maudsley, & Martin, 2012), a reduction in clinical seizure activity, improved energy metabolism, and a reduction in stress‐induced biochemical effects within the hippocampus, thus improving the internal environment and improving cognitive and behavioral function (Brownlow, Jung, Moore, Bechmann, & Jankord, 2017).

In addition, common adverse reactions of KD treatment include vomiting, diarrhea, constipation, and sleep. A small percentage of children will experience asymptomatic hypoglycemia, liver dysfunction, urinary stones, and other adverse reactions; however, the adverse reactions have no effect on normal growth and development of children. The results of this study showed that 23 children had 1–3 types of adverse reactions during KD treatment, of whom 20 had varying severity of digestive symptoms, and 4 had sustained symptoms. In all, 11 patients had asymptomatic hypoglycemia, and 12 had increased sleep needs within 1 week of onset, and 1 had abnormal liver function. After symptomatic treatment, the adverse reactions in most patients subside, which was in agreement with the findings of other domestic and international researchers.

A number of researchers may be more concerned about how the long‐term treatment with a KD will affect the growth curve, resulting in application concerns. Numis, Yellen, Chu‐Shore, Pfeifer, and Thiele (2011) reported that the growth curve is not affected by the KD, but three females with GLUT1 deficiency who were treated with a KD for 5 years showed that the bone mineral content and bone mineral density were not negatively affected (Bertoli et al., 2014). Adverse reactions should be given full attention, and effective measures should be taken for prevention and/or intervention.

In short, KD therapy plays an important role in the treatment of pharmacoresistant epileptic encephalopathy, and is important for the treatment of children with epileptic encephalopathy treatment. The results of this study suggest that KD not only has good clinical efficacy in the treatment of pharmacoresistant epileptic encephalopathy in children, but also can significantly reduce the interictal epileptic discharge frequency and improve the background rhythm of EEGs. The early decrease in the epileptic discharge index during the sleep period is beneficial for decreasing epileptic seizures in patients with epileptic encephalopathy, thus improving the efficacy, and in reducing the clinical seizures at the same time, improving cardiac function, and improving cognitive function, language, and motor function, thereby enhancing the quality of life of patients. Although some adverse effects exist during KD treatment, the effects are mainly transient or treatable gastrointestinal symptoms and metabolic disorders, but all adverse reactions were mild, and all can be tolerated and improved through appropriate treatment and adjustment. For children with pharmacoresistant epileptic encephalopathy, KD treatment is regarded as a clinically effective treatment option when the regular combination of a variety of antiepileptic drugs are ineffective, so KD treatment is worthy of wide application by pediatric neurologists.

## CONFLICT OF INTEREST

The authors declare that they have no conflict of interest.
